# Transferability and Polymorphism of SSR Markers Located in Flavonoid Pathway Genes in *Fragaria* and *Rubus* Species

**DOI:** 10.3390/genes11010011

**Published:** 2019-12-21

**Authors:** Vadim G. Lebedev, Natalya M. Subbotina, Oleg P. Maluchenko, Tatyana N. Lebedeva, Konstantin V. Krutovsky, Konstantin A. Shestibratov

**Affiliations:** 1 Pushchino State Institute of Natural Sciences, Prospekt Nauki 3, 142290 Pushchino, Russia; vglebedev@mail.ru (V.G.L.); natysubbotina@rambler.ru (N.M.S.); 2 Branch of the Shemyakin-Ovchinnikov Institute of Bioorganic Chemistry, Russian Academy of Sciences, Prospekt Nauki 6, 142290 Pushchino, Russia; schestibratov.k@yandex.ru; 3 All-Russian Research Institute of Agricultural Biotechnology, Timiriazevskaya Str. 42, 127550 Moscow, Russia; oleg.maluchenko@mail.ru; 4 Institute of Physicochemical and Biological Problems of Soil Science, Russian Academy of Sciences, Institutskaya Str. 2, 142290 Pushchino, Russia; tanyaniko@mail.ru; 5 Department of Forest Genetics and Forest Tree Breeding, Georg-August University of Göttingen, Büsgenweg 2, 37077 Göttingen, Germany; 6 Center for Integrated Breeding Research, Georg-August University of Göttingen, Albrecht-Thaer-Weg 3, 37075 Göttingen, Germany; 7 Laboratory of Population Genetics, N. I. Vavilov Institute of General Genetics, Russian Academy of Sciences, Gubkin Str. 3, 119333 Moscow, Russia; 8 Laboratory of Forest Genomics, Genome Research and Education Center, Institute of Fundamental Biology and Biotechnology, Siberian Federal University, 660036 Krasnoyarsk, Russia; 9 Department of Ecosystem Science and Management, Texas A&M University, 2138 TAMU, College Station, TX 77843-2138, USA

**Keywords:** *Fragaria*, *Rubus*, microsatellites, transferability, polymorphism, introns, exons, flavonoid biosynthesis pathway, transcription factor genes, chitinase

## Abstract

Strawberry (*Fragaria*) and raspberry (*Rubus*) are very popular crops, and improving their nutritional quality and disease resistance are important tasks in their breeding programs that are becoming increasingly based on use of functional DNA markers. We identified 118 microsatellite (simple sequence repeat—SSR) loci in the nucleotide sequences of flavonoid biosynthesis and pathogenesis-related genes and developed 24 SSR markers representing some of these structural and regulatory genes. These markers were used to assess the genetic diversity of 48 *Fragaria* and *Rubus* specimens, including wild species and rare cultivars, which differ in berry color, ploidy, and origin. We have demonstrated that a high proportion of the developed markers are transferable within and between *Fragaria* and *Rubus* genera and are polymorphic. Transferability and polymorphism of the SSR markers depended on location of their polymerase chain reaction (PCR) primer annealing sites and microsatellite loci in genes, respectively. High polymorphism of the SSR markers in regulatory flavonoid biosynthesis genes suggests their allelic variability that can be potentially associated with differences in flavonoid accumulation and composition. This set of SSR markers may be a useful molecular tool in strawberry and raspberry breeding programs for improvement anthocyanin related traits.

## 1. Introduction

The Rosaceae family comprises approximately 3000 species and includes very important fruit, berry, and ornamental plants. This family has been relatively recently reorganized into three subfamilies: Dryadoideae, Spiraeoideae, and Rosoideae. The latter one includes cultivated berries in the genera *Fragaria* (strawberry) and *Rubus* (raspberry and blackberry) [1]. Strawberry and raspberry are in especially high demand among consumers due to their appearance, taste, and aroma [2,3]. They are also rich in antioxidants and other bioactive compounds beneficial for human health. The strawberry is the most consumed berry worldwide—more than 9 million tons were harvested in 2017, while the production of raspberry and blackberry increased by 50% for the period 2010–2017 and exceeded 800,000 tons [4]. It is suggested that the consistent demand for healthy and delicious berryfruit observed in the current decade will increase in the nearest future [2].

Recently, the high interest in berry crops has led not only to increased production levels, but also to the expansion of *Rubus* and *Fragaria* breeding programs; several dozens of them are now known [3,5]. For a long period of time, the main directions in breeding were crop yield and disease resistance, but in the past years, fruit sensorial and nutritional qualities have become major objectives [6]. However, *Rubus* and *Fragaria* breeding is complicated because of several genetic problems including polyploidy and the highly heterozygous nature of the germplasm [3,5]. The genus *Rubus* consisted of several hundred species, and the ploidy level can widely vary among them. Raspberries are mainly diploids (2*n* = 14), while blackberries may vary from diploids to dodecaploids (2*n* = 84), whereas the hybrids between them can be hexaploid (loganberry) or septaploid (boysenberry) [7]. A total of 22 wild species of *Fragaria* have been described. Almost half of them are diploids (2*n* = 14), while tetra-, hexa-, octo-, and decaploid species are also known [8]. The main cultivated strawberry crop, *F*. × *ananassa*, is a hybrid between *F. chiloensis* and *F. virginiana*, but the origin of its octoploid genome from four diploid ancestors has long been unknown. In addition to *F. vesca* and *F. iinumae*, contribution of different species was assumed, but only in 2019 the phylogenetic analyses of Edger et al. [9] provided a strong genome-wide support that *F. iinumae*, *F. nipponica*, *F. viridis*, and *F. vesca* are diploid progenitor species. *F. vesca* (the wild strawberry) and *F. viridis* (the green strawberry) can be used in strawberry breeding as donors of abiotic and biotic stress resistance and fruit aroma [10] and firm flesh, remontant flowering habit, and an acidic apple-like aroma [11], respectively.

Developing a new cultivar by traditional methods is a very long process that can take up to 15 years for raspberry [5] or 10 years for strawberry [12]. Molecular markers, however, can be used at any stage of plant growth and can increase the speed and accuracy of germplasm assessment. A good choice for breeding purposes is a simple sequence repeat (SSR) or microsatellite markers consisting of tandem repeats (1–6 nucleotides). Due to their codominant inheritance, high level of polymorphism, and abundance in genome, they play an important role in identifying genomic regions associated with the traits of economic importance [13]. SSR markers for the *Rubus* and *Fragaria* species were first developed in the early 2000s [14,15] and in subsequent years, a number of studies were carried out, including evaluation of marker transferability. SSR transferability depends on genetic distance between individual specimens. SSRs are more transferable, overall, within the species of the same genus or among related genera within families than between remote genera and different families [16]. The *Fragaria* and *Rubus* species from Rosoideae subfamily have both the same basic number of chromosomes (*x* = 7) and close phylogenetic relationships based on their chloroplast and nuclear DNA markers, as well as similar morphology. These facts suggest collinearity between *Fragaria* and *Rubus* genomes [17]. In raspberry breeding, interspecific hybridization is widely used, and development of molecular genetic markers that can be transferred between different species, especially with different ploidy, becomes an important task. However, very few molecular genetic markers are known for *Rubus*, and fewer are transferable between species [13]. All strawberry cultivars now available at the market have been produced using traditional breeding methods [3]. Among raspberry cultivars, there are currently only two productive cultivars with root rot resistance that have been produced using the marker-assisted selection (MAS), but they are still in the commercial trial stage [18]. The development of new molecular genetic markers that could be used for molecular genetic characterization of both wild species and germplasm would accelerate the breeding of new cultivars [2]. The transferability is very important for their wide use.

The SSR markers can be developed using either genomic or expressed sequence tag (EST) nucleotide sequences. The EST-based markers are more transferable and more useful in MAS as they are linked with expressed genes and could be associated with important agronomical traits [19]. The strawberry studies have shown that EST-SSR markers were more transferable compared to random genomic nongenic noncoding SSR (ncSSR) markers [20,21]. At the same time, it is known that ESTSSR markers are generally less polymorphic than ncSSRs that mostly represent noncoding genomic regions because of a greater DNA sequence conservation of transcribed regions [22]. There are no markers that would be universal or ideal for all practical applications and tasks. For some tasks such as analyses of population structure, genetic drift, migration, gene flow, mating system, and individual identification of clones, cultivars, etc. selectively neutral ncSSRs would be the most appropriate markers, but for functional analysis of adaptive variation, MAS, QTL mapping, etc., ESTSSRs that represent functional alleles and haplotypes and link to important adaptive and breeding traits would be more appropriate and useful markers. Among SSR markers, those that are located in introns and untranslated regions (UTRs) are more polymorphic and potentially can combine advantages of both ncSSR and EST-SSR markers, while those that are located in exons are less polymorphic, but more likely to be under direct selection or represent selectively different alleles, and therefore are more useful for MAS because they might better represent functional traits important for breeding. We studied diploid *Rubus* species and confirmed that SSR markers located in introns were more variable in comparison to EST-SSR located in exons [23]. However, development of such markers complete nucleotide sequences of important adaptive and breeding trait related genes.

**Figure 1:**
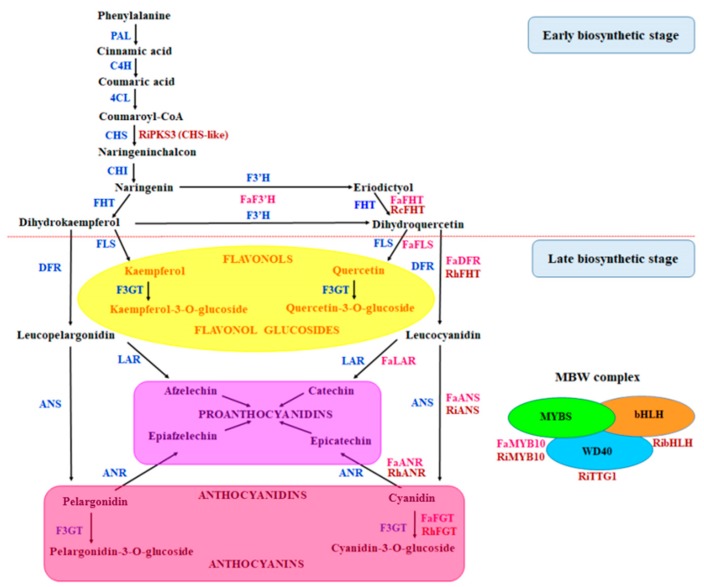
Schematic representation of flavonoid biosynthesis in strawberry and raspberry. PAL—phenylalanine lyase; C4H—cinnamate 4-hydroxylase; 4CL—4-coumarate CoA ligase; CHS—chalcone synthase; CHI—chalcone isomerase; F3’H—flavonoid 3’-hydroxylase; FHT—flavonone 3-hydroxylase; FLS—flavonol synthase; DFR—dihydroflavonol 4-reductase; LAR—leuco-anthocyanidin reductase; ANS—anthocyanidin synthase; ANR—anthocyanidin reductase; F3GT—flavonoid 3-O-glycosyl transferase. Pink color indicates strawberry genes and dark-red color indicates *Rubus* genes used in this study.

Many MAS studies are aimed at developing markers representing key genes, but only few studies have focused on the developing markers representing structural and regulatory genes of metabolic pathways in cultivars with contrasting phenotypic traits of interest [24]. We are especially interested in developing SSR markers representing flavonoid pathway genes because the main polyphenols in fruits are flavonoids (Figure 1). Complete nucleotide sequences are already available for many of these genes. Thus, breeding of new berry cultivars with the improved nutritional value using MAS and SSR markers representing flavonoid biosynthesis-related genes seems to be a highly promising approach. The combinatorial interactions of the regulatory genes with structural genes that act to control the flux of various branches of the pathway ultimately determines the flavonoid composition [25]. However, as far as we know, SSR markers representing *Fragaria* and *Rubus* transcription factors (TFs) were not developed before our study. Main aims of our study were to: (1) develop SSR markers using coding (CDS) and non-coding (NCDS) sequences of structural and regulatory genes of flavonoid biosynthesis pathways, (2) evaluate them in *Rubus* and *Fragaria* species of different ploidy, (3) test cross-species transferability within and among *Rubus* and *Fragaria* genera, (4) assess the relationship of transferability and polymorphism of SSR markers with location of primer binding sites for polymerase chain reaction (PCR) and SSR loci in respective genes. Some of these genes could control anthocyanin content that correlates strongly with color. Therefore, strawberry and red raspberry cultivars with a wide range of berry colors were used in our study. In addition to nutritional value, disease resistance is also considered to be a valuable trait for the berry crops. The key factor for developing pathogen resistance in new cultivars is the identification and introgression of genes from cultivated varieties or their wild relatives [26]. Strawberry and raspberry production suffers from a number of agriculturally important diseases, and, therefore, we included in our study developing of SSR markers in strawberry genes encoding pathogenesis-related (PR) proteins – β-1,3-glucanases (PR-2 family) and chitinases (PR-3, 4, 8, 11 families). Since the sequences for raspberry chitinase genes were absent in the NCBI GenBank database, we sequenced the fragments of chitinase III genes in raspberry cultivars.

## 2. Materials and Methods

### 2.1. Plant Materials

Sixteen *Fragaria* specimens, including *F*. × *ananassa*, *F. vesca*, *F. viridis* and (*F*. × *ananassa*) × *Comarum palustre*, and 32 *Rubus* cultivars, including red raspberry (*R. idaeus*; Idaeobatus subgenus), black raspberry (*R. occidentalis*; Idaeobatus subgenus), blackberry (*Rubus* subgenus), cloudberry (*R. chamaemorus*; Chamaemorus subgenus), arctic bramble (*R. arcticus*; Cyclastis subgenus), and hybrid arctic bramble (*R. × stellarcticus*; Cyclastis subgenus) were chosen to genotype newly developed SSR loci located in the flavonoid biosynthesis and pathogenesis-related genes. These cultivars have a wide range of fruit color, ploidy and various geographic and genetic origins, but mostly of Russian origin (Table 1). The plants used in this study were kindly provided by I.A. Pozdniakov (OOO Microklon, Pushchino, Russia). Each cultivar represented a microclonally vegetatively propagated line containing genetically identical plants. Therefore, a single specimen per culture was used for further DNA isolation and genotyping.

### 2.2. Simple Sequence Repeat (SSR) Marker and Polymerase Chain Reaction (PCR) Primer Development

The WebSat software [27] was used to detect SSR loci in the nucleotide sequences of *F*. × *ananassa* and *Rubus* genes available in the NCBI GenBank database (http://www.ncbi.nlm.nih.gov) (Table S1). To search for SSRs, the following threshold criteria were used: ten for mononucleotide repeats, five for dinucleotide motifs, four for tri-, three for tetra-, and two for penta-, and hexanucleotide repeats. The Primer 3 software (http://primer3.org) was used to design appropriate (PCR) primers based on the sequences flanking the SSR loci.

Primers were designed using the following criteria: primer length of 18–27 bp (optimally 22 bp), GC content of 40–80%, annealing temperature of 57–68 °C (optimally 60 °C), and expected amplified product size of 100–400 bp. Primers for the *RiG001* locus were as in [28]. Primers were synthesized by Syntol Comp. (Moscow, Russia) and are presented in Table S1.

### 2.3. DNA Isolation, PCR Amplification and Fragment Analysis

A single DNA sample per each specimen was produced from young expanding leaves representing a single plant per each sample. Total genomic DNA was extracted using the STAB method [29]. The quality and quantity of extracted DNA were determined by the NanoDrop 2000 spectrophotometer (Thermo Fisher Scientific Inc., Waltham, MA, USA). The final concentration of each DNA sample was adjusted to 50 ng/µL in TE buffer before the PCR amplification.

For genotyping, PCR was performed separately for each primer pair using a forward primer labeled with the fluorescent dye 6-FAM and an unlabeled reverse primer (Syntol Comp., Moscow, Russia). The PCR amplification was performed in a total volume of 20 µL consisted of 50 ng of genomic DNA, 10 pmol of the labeled forward primer, 10 pmol of an unlabeled reverse primer, and PCR Mixture Screenmix (Evrogen JSC, Moscow, Russia). After an initial denaturation at 95 °C for 3 min, DNA was amplified during 33 cycles in a gradient thermal cycler (Bio-Rad Laboratories, Inc., Hercules, CA, USA) programmed for a 30 s denaturation step at 95 °C, a 20 s annealing step at the optimal annealing temperature of the primer pair, and a 35 s extension step at 72 °C. A final extension step was done at 72 °C for 5 min.

The PCR generating clear, stable, and specific DNA fragments within an expected length (200–400 bp) were considered as successful PCR amplifications. If a primer pair failed three times to amplify template DNA that was amplified with other primers, then it was scored as a null genotype.

Separation of amplified DNA fragments was performed in an ABI 3130xl Genetic Analyzer using S450 LIZ size standard (Syntol Comp.). Peak identification and fragment sizing were done using the Gene Mapper v4.0 software (Applied Biosystems, Foster, CA, USA).

### 2.4. Genetic Data Analysis

Genetic statistics were calculated for each polymorphic microsatellite marker. The number of alleles, observed (*H_o_*) and expected (*H_e_*) heterozygosities, and polymorphic information content (PIC) for 32 diploid *Rubus* cultivars were calculated using the PowerMarker 3.25 software [30]. Analogous parameters for 13 octoploid *Fragaria* cultivars were calculated using the GenoDive 3.0 software [31]. Principal component analysis (PCA) and construction of the box plots were performed with the PCORD 5 software [32].

### 2.5. Chitinase Gene Sequencing and Sequence Alignment

Based on the expected homology between *Fragaria* and *Rubus* species, the following two primers were used for PCR amplification of a 528 bp long fragment homologous to the strawberry chitinase III gene in three raspberry cultivars: Ch-Up1 5′-GAAGATGCCCGCCAAGTTG and Ch-Low2S 5′-TTGATGGAGGAGCTGTATC. The amplification reaction mixture (25 µL) contained ~0.15 µg of genomic DNA, ScreenMix-HS buffer (Evrogen JSC, Moscow, Russia), 0.8 mM of each primer, and Milli-Q water. The PCR protocol included an initial denaturation step at 95 °C for 5 min followed by 31 cycles consisting of 45 s at 95 °C, 30 s at 59 °С, and 60 s at 72 °C each. A final step of 10 min at 72 °C ended the cycles followed by a hold at 4 °С. The PCR products were purified and sequenced by Evrogen JSC. The chitinase III sequences were aligned, visualized and manually inspected using the MView 1.63 software (www.ebi.ac.uk/Tools/msa/mview).

## 3. Results

### 3.1. Development and Characterization of SSR Markers

A total of 118 SSR loci were detected in 21 gene sequences (45.6 Kb). The number of SSRs ranged from one to 13 per gene (5.6 on average). One SSR was found per every 387 bp on average; less frequent in exons with one SSR per every 628 bp, but more frequent in introns with one SSR per every 263 bp on average. In our SSR analysis, loci with pentanucleotide motifs were detected at the highest frequency (45%), followed by loci with hexa-(25%) and dinucleotide (13%) motifs. Loci with tetra-, mono- and trinucleotide motifs were relatively less frequent—8%, 5%, and 4%, respectively. All loci with mononucleotide repeats consisted of only T nucleotide and contained from 10 to 12 T nucleotides. Among 17 loci with dinucleotide repeats, the AT/TA motif was the most frequent (47%), followed by CT/GA (24%) and GT/CA (18%). On average, number of repeats were 9.7 for loci with dinucleotide motifs and 7.5 for loci with trinucleotide motifs ranging from 5 to 32 and 4 to 14 motifs per locus, respectively. Loci with tetranucleotides motifs contained only three repeats, and loci with penta- and hexanucleotide motifs contained only two repeats.

Location of microsatellite loci in CDS (exons) and NCDS (introns, 5′ and 3′UTRs, and upstreams—upfront regions further than 500 bp from the first exon) was determined, with majority of them (35%) being located in introns, 28% in exons, 14% in 5′UTRs and upstreams, and 9% in 3′-UTRs (Table 2). Loci with pentanucleotide repeats prevailed everywhere (41–55%), except in upstreams (29%), where the proportion of hexanucleotide SSRs was higher (41%). A relatively high proportion of hexanucleotide SSRs was also in exons (33%). The nucleotide distribution was approximately equal in the exons (21, 24, 26, and 28% for T, G, A, and C, respectively), while in NCDS T (46%) and A (34%) prevailed over C (12%) and G (8%).

However, not all microsatellite loci could be developed into useful SSR markers. For example, some microsatellite loci were located at the end of the sequenced DNA fragment. Microsatellite loci mononucleotide repeats were also not used for developing SSR markers. We also tried to use various combinations of the location of SSR loci and annealing sites for PCR primer pairs. This analysis resulted to selection of 24 sequences ranging from 122 to 400 bp long and harboring 43 microsatellites. Finally, 24 primer pairs were successfully designed (Table S1). In addition to the newly developed markers, we used the *RiG001* marker from *R. idaeus* [28]. The developed SSR loci were in all gene regions except 3′UTRs: ten were in introns, eight in exons, two in upstreams, and one was in 5′UTR (Table S1). In addition, four loci were located at the junction of CDS and NCDS (two loci at the junction of 5′UTR and exon, and intron and exon each). Eleven markers contained more than one SSR locus. The *FaFS01*, *FaAR01*, *RhUF01*, *FaMY02*, and *FaFG01* markers contained two SSR loci, the *FaCH01*, *FaCH02*, *FaF3H01*, *FaBG01*, and *RiMY01* markers contained three SSR loci, and the marker *FaMY01* contained five SSR loci. Among the new SSR markers developed, 15 were developed using *F*. × *ananassa* sequences whereas nine were developed using *Rubus* species sequences.

### 3.2. Cross-Specific Transferability of SSR Markers

The 24 genic SSRs developed in this study and one published SSR from *R. idaeus* (Table S1) were evaluated for cross-amplification in two important genera of the subfamily Rosoideae, *Fragaria* and *Rubus*. These 25 SSR loci represented 18 structural and regulatory flavonoid biosynthesis genes and three PR protein genes. A total of 48 specimens belonging to 11 species and hybrids with a wide range of ploidy (di-, tetra-, hexa-, hepta- and octoploids) were used for marker validation (Table 1). The collection of selected specimens included samples from different breeding programs worldwide, including specimens from Finland, Germany, The Netherlands, Poland, Russia, Sweden, Switzerland, UK, Ukraine, and USA. Cross-amplification results and allele sizes are presented in Table S1. Twenty two of the 25 primer pairs amplified a PCR product or products of approximately the size expected for a homologous gene. Only primer pairs for the *RiMY01* locus generated multiple bands of approximately the expected size in *R. × stellarcticus* hybrids. In total, 10 primer pairs, representing nine out of 21 genes, amplified a product of the expected size in all two genera, indicating that primer binding sites were conserved across two rosaceous genera screened.

All primer pairs (15) from *F*. × *ananassa* amplified a PCR product in each of the 14 strawberry specimens including *Fragaria* × *Comarum* hybrid that showed 100% transferability despite the various genetic origin. More than two (up to eight) fragments were amplified in the *F*. × *ananassa* specimens, which was expected because of their polyploid nature. Ten SSR markers (67.7%) revealed genetic differentiation among strawberry specimens, while polymorphism was not detected in five SSR markers. Among them, two loci (*FaAR01* and *FaCH01*) each had two amplified fragments, but they were identical in all tested specimens. In order to evaluate the transferability of SSR markers in the diploid *Fragaria* species, the developed PCR primer pairs were used to genotype *F. vesca* and *F. viridis*. 

Transferability of SSRs within *Fragaria* was high. Eleven of these primers (73.3%) amplified fragments in *F. vesca* and *F. viridis*. However, four strawberry primer pairs for the *FaDR01*, *FaLR01*, *FaMY01*, and *FaMY02* loci failed to amplify in two *Fragaria* species, suggesting that either these sequences have diverged between octoploid and diploid species or are not present in *F. vesca* and *F. viridis*, but are present in two other ancestors of the octoploid genome of *F*. × *ananassa*.

The transferability of *Rubus* markers within the genus was lower than that of *Fragaria—*50–70% for 10 markers, depending on the species. The maximum transfer was in red raspberry, the minimum—in hybrid arctic bramble: four markers worked in all six hybrid arctic bramble cultivars, but *RcFH01*—only in two of them. All of the five *Rubus idaeus*-derived SSR markers successfully amplified fragments in all red raspberry cultivars. In addition, a marker from *R. coreanus* and only one markers from blackberry (*RiMY01*) were amplified in red raspberry cultivars. Six *Rubus* SSRs amplified PCR products in all *Rubus* species and five in all six hybrid arctic bramble cultivars. In addition, the *RiG001* marker was amplified only in red raspberry. Typically, markers are amplified within the same species, but three markers *RhDR01*, *RhDR02*, and *RhAR01* developed originally in a blackberry (cultivar Arapoho, NCBI GenBank accession number JF764809) did not produce a product in our blackberry cultivars and hybrids. Moreover, these three markers were not amplified in any tested specimen.

Transferability from *F*. × *ananassa* to *Rubus* species was demonstrated for 5–7 out of the 15 primer pairs (33.3–46.7%). Thus, the transferability of strawberry markers decreased as cultivars become less related: all 15 markers were amplified in *F*. × *ananassa*, 11 markers in two diploid *Fragaria* species, and 5–7 in *Rubus* species. Six out of the 15 *F*. × *ananassa* primer pairs (40%) amplified fragment of the expected size in red raspberry and hybrid cultivars such as Loganberry, Tayberry, and Boysenberry. Successful cross amplification in other *Rubus* species ranged from 33.3% in black raspberry and blackberry to 46.7% in Nordic species (*R. chamaemorus*, *R. arcticus*, and *R. × stellarcticus*), where the *FaLR01* marker was also amplified, while it did not produce any PCR product in other *Rubus* species. The majority of *Rubus* cultivars had one or two amplified fragments per primer pair, however, for some polyploidy cultivars, as well as the blackberry and hybrids, there were more than two fragments (up to four) amplified by some primer pairs. The octoploid species *R. chamaemorus* almost always showed the presence of only one or two fragments.

Transferability of *Rubus* SSRs to the *Fragaria* species was relatively low: only three out of 10 markers (all from *R. idaeus*) were amplified, and there were no differences among species. In total, four out of 14 SSR markers had amplified fragments in *F. vesca* and *F. viridis* in the same range of size as that in *F*. × *ananassa*, and the rest can be used to separate diploid species and octoploid strawberry. Six SSRs had *F*. × *ananassa* and unique fragments amplified, and four markers only unique fragments: the same for two diploid species (*FaFH01*) and different (*FaCH01*, *FaBG01*, and *RiMY01*). Only the *FaFS01* marker amplified two fragments unique to *Fragaria* × *Comarum* hybrid.

Twelve out of 18 SSR markers (66.7%) were found to be polymorphic in the 13 strawberry specimens. In addition, three markers (*FaAR01*, *RiAS01*, and *FaCH01*) had the same two fragments amplified in all specimens. Thus, these markers were monomorphic in these specimens, but may be polymorphic in a wider collection. The majority of the polymorphic SSR markers in *Fragaria* genes (7 out of 10) had more than two fragments amplified in strawberry specimens, probably, originating from four genomes of octoploid strawberry. In total, 11 out of 14 markers (78.6%) were polymorphic in the genus *Rubus*. However, only one of them (*RhUF01*) was polymorphic in all species. There are two reasons for this, firstly, not all markers were amplified in all species. Secondly, a small number of specimens were used for most species. Most of the markers were polymorphic in species with a large number of tested specimens and/or having a hybrid origin. In red raspberry (14 cultivars), hybrid Arctic bramble (six specimens), and *Rubus* hybrid (four specimens) the proportion of polymorphic markers was 61.5%, 58.3% and 66.7%, respectively. This approximately corresponds to the proportion of polymorphic markers in strawberry. In black raspberry and arctic bramble, each having two specimens tested, 27.3% and 30.8% markers were polymorphic, respectively.

Monomorphic markers of strawberry *FaFS02*, *FaTG01* and *FaCH01* produced alleles of the same size in *Fragaria* and *Rubus* species—271, 324, and 324 + 329 bp, respectively. In addition, the polymorphic strawberry markers *FaFS01*, *FaLR01*, and *FaFH01* had one allele amplified in northern *Rubus* species, which was almost the same (different by only one or two nucleotides) as one of the main strawberry alleles. Interestingly, the alleles of *Rubus* species for seven markers from *Fragaria* had almost the same size as expected or were different by no more than 10–20 nucleotides in both directions, while the alleles in strawberry for *Rubus* markers (*RiAS01*, *RiMY01*, and *RiHL01*) were always less than the expected size by about 40–100 nucleotides. The most multi-allelic SSR markers (17–19 alleles; *FaFS01*, *FaMY0*2, and *RiMY01*) contained dinucleotide repeats in introns and presented series of consecutive alleles in 2-bp steps. In addition, the *RiAS01* marker with exon-located locus showed a number of alleles with a step of 24: 261, 285, 309, 333, and 357 in *Rubus* species; 261, 285, and 333 in *Fragaria* species.

### 3.3. Allelic Polymorphism and Genetic Diversity

Number of alleles, expected (*H_e_*) and observed (*H_o_*) heterozygosities, and polymorphism information content (PIC) were calculated for 12 polymorphic SSR markers in 13 octoploid strawberry specimens (Table 3) and in 24 diploid *Rubus* cultivars (Table 4). The number of alleles in strawberry specimens varied widely among these markers ranging from two in *RiMY01* to 14 in *FaFS01*, with 6.7 on average, respectively. The *H_o_* and *H_e_* values ranged from 0.37 to 0.90 and 0.35 to 0.90, with 0.63 and 0.66 on averages, respectively. The *FaCH02* locus, located in exon, demonstrated the lowest heterozygosity. The PIC ranged from 0.34 to 0.89 with an average of 0.66. The most markers (nine out of 12) had PIC values higher than 0.5, suggesting that these markers can efficiently measure genetic diversity in strawberry.

From two alleles in the *RiHL01* marker to 13 alleles in the *RiMY01* marker were amplified in 24 diploid *Rubus* cultivars, with a mean number of 4.6 alleles per locus for 10 polymorphic markers (Table 4). Observed (*H_o_*) and expected (*H_e_*) heterozygosity ranged from 0 to 0.5 and 0.04 to 0.84 with mean values of 0.23 and 0.51, respectively.

Unexpectedly, the lowest heterozygosity was observed in the intron-located *RiHL01*marker. The PIC ranged from 0.04 to 0.82 with a mean of 0.46, which was noticeably lower than in strawberries (0.66), and only six markers had PIC values higher than 0.50 suggesting their high potential to measure high genetic diversity. The other four markers had *PIC* ranging from 0.25 to 0.50 showing their rather moderate potential to measure genetic diversity, and only the *RiHL01* marker was slightly informative (<0.25).

**Figure 2:**
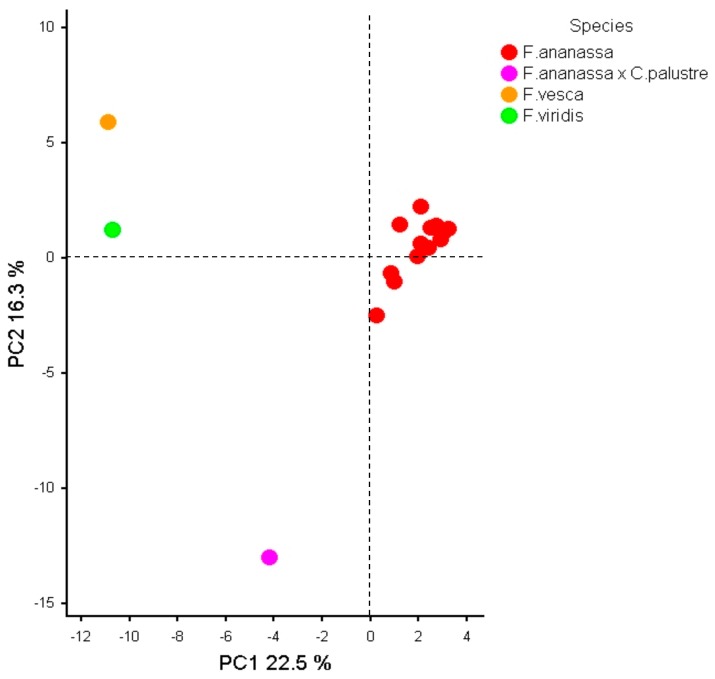
PCA of genotyped strawberry (*Fragaria*) species and specimens based on 13 polymorphic SSR markers.

**Figure 3:**
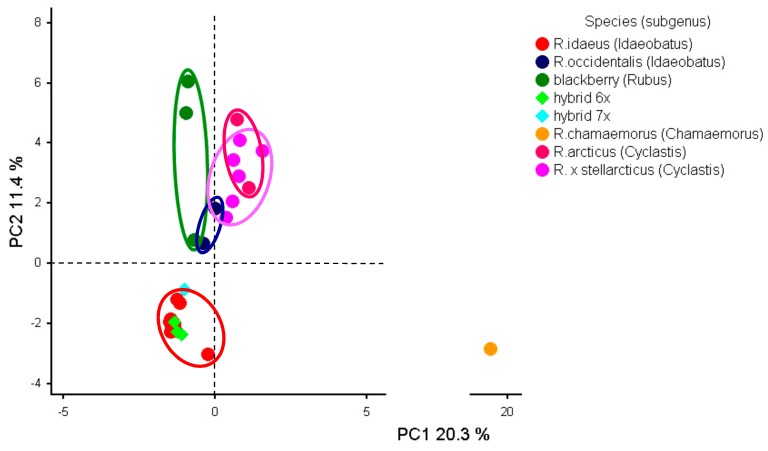
PCA of *Rubus* cultivars based on 10 polymorphic SSR markers.

Principal Component Analysis (PCA) was used to reveal genetic relations among *Fragaria* (Figure 2) and *Rubus* (Figure 3) species and cultivars based on SSR markers representing the flavonoid biosynthesis pathway genes. The first three PCs explained 22.5%, 16.3%, and 9.8% of the total variance in *Fragaria*, respectively (Figure 2). All cultivars of *F*. × *ananassa* formed a compact group and were completely separated from diploid *Fragaria* species and (*F*. × *ananassa*) × *C. palustre* hybrid. *F. vesca* and *F. viridis* were grouped along the PC1, whereas *F. virids* and *F*. × *ananassa* along the PC2. Interestingly, *Fragaria* × *Comarum* hybrid (*F*. × *ananassa*) × *C. palustre* was very distant from the rest *Fragaria* cultivars and species, which is in agreement with a pink color of flowers in this hybrid, which makes it also very different from other cultivars. The PC3 did not contribute much in delineation of cultivars, therefore plots with PC3 are not presented here.

The PCA in Figure 3 represents the relationships between 32 individual *Rubus* cultivars and species. The first three PCs explained 20.3%, 11.4%, and 9.7% of the total variance, respectively. The PC3 did not contribute much in delineation of cultivars, therefore plots with PC3 are not presented here. In general, the grouping was as expected, and a good discrimination was observed between four *Rubus* subgenera—all of them were well-separated along PC1 and PC2. Unexpectedly, arctic bramble (*R. arcticus*; Cyclastis subgenus) was very distant from other *Rubus* species. There was a clear overlap between two *R. arcticus* and *R. × stellarcticus* clusters. Despite belonging to different subgenera black raspberry (*R. occidentalis*; Idaeobatus subgenus) cultivars were closer to blackberry (*Rubus* subgenus), which is in agreement with having also a common black color of their berries. The *Rubus* hybrids cluster coincided with the red raspberry (*R. idaeus*; Idaeobatus subgenus) cluster and was clearly distinguished from blackberry group, which suggests closer relationship of hybrids with red raspberry. All these hybrids have berries in different shades of red color.

### 3.4. Genetic Data Analysis

To determine the relationship between transferability and polymorphism of SSR markers and the location of loci and of primer pairs, the data were grouped in the Table 5. The identification of the location of the primer binding sites and the separation of the SSR markers into four groups based on this trait showed their clear connection with transferability level. When both primers are located in the conserved exons, the complete transferability is observed both within *Fragaria* and *Rubus* species and the cross-amplificatons between the *Fragaria* and *Rubus* genera. In the opposite direction (from *Rubus* to *Fragaria*), only one out of three markers (*RiMY01*) was amplified.

When one of the primer binding site is located in more variable NCDS (introns or 5′UTR), the transferability level decreases. With intrageneric transferability, two of the *F*. × *ananassa* markers, *FaMY01* and *FaLR01*, were no amplified product in the diploid *F. vesca* and *F. viridis*, while out of the three *Rubus* markers, only the *RiHL01* (representing TF) marker was amplified in all *Rubus* cultivars. The *RiG001* marker was amplified only in red raspberry, and *RcF3H* was transferred in Anna and Beata, but not in Linda, Sofia, Astra, and Aura hybrid arctic bramble cultivars. The transferability between the genera was also much lower. Out of the five *Fragaria* markers, only *FaLR01* was amplified in the Nordic species: cloudberry, Arctic bramble, and hybrid Arctic bramble, and *FaCH01*—in some cultivars of red raspberry, hybrids and Nordic species, but was not amplified in black raspberry and blackberry at all. A similar situation was observed when both primer binding sites were located in NCDS: the amplification of some markers failed in *Fragaria* diploid species, and none of the *Fragaria* markers was amplified in *Rubus*.

The location of one of the binding sites across intron-exon junction had the worst effect on transferability. Three out of four markers were not amplified in any specimen, even when the second primer site was located in exon and in the same species (blackberry). Only the *RiMY01* marker amplified some fragments, but it was inconsistent; multiple fragments were generated in Anna and Beata, but no fragments in Linda, Sofia, Astra, and Aura hybrid Arctic bramble cultivars.

We also observed a clear relationship between the location of loci and polymorphism level. When loci were located in introns or 5′UTR, the larger number of alleles, up to 14 alleles in *F*. × *ananassa* and 17 alleles in *Rubus*, was observed, and all markers were polymorphic. On the other hand, the allele number in exon-located loci did not exceed four in *F*. × *ananassa* (*FaCH02*) and five in *Rubus* species (*RiAS01*, *RhUF01*) and many markers were monomorphic. For example, all three markers that amplified one fragment in strawberry specimens were located in exons. Markers that contained more than one microsatellite locus located both in CDS and NCDS demonstrated high polymorphism (*FaFS01*, *FaFG01*) as well as monomorphism (*FaCH01*). In total, nine out of 12 polymorphic markers in strawberry were in NCDS, two in exons + introns, and only one in exon (*FaCH02*). More than half (six out of 11) of polymorphic markers in *Rubus* were also located in introns. With regard to the relationship between polymorphism and SSR motif type, the highest allelic variation was revealed in markers with dinucleotide motifs and large number of repeats, such as in the *FaMY02*, *FaFS01*, and *RiMY01* markers. It should be noted that two of these markers represented the *MYB10* TF genes in raspberry and strawberry.

### 3.5. Sequence Analysis of Chitinase III Genes

Based on the *F*. × *ananassa* chitinase III (chi3) sequence (GenBank accession number AF134347), we designed a pair of primers and amplified cDNA fragments from three raspberry cultivars with yellow-, orange- and red-colored berries (Zolotaya Osen, Oranzhevoe Chudo, and Babye Leto II, respectively). Sequencing confirmed that these fragments were composed of 528 nucleotides within the full length of open reading frame (ORF) and encoded 176 amino acids. The red-fruited Babye Leto II cultivar differed from the other two cultivars by two synonymous nucleotide substitutions. The nucleotide sequences of three chitinase III gene fragments were deposited in the NCBI GenBank (accession numbers MK333194, MK333195, and MK333196, respectively). The translated amino acid sequences of the raspberry chitinase III were aligned with published sequences of strawberry (cv. Chandler) and raspberry of unknown origin (Figure S1) [33]. The identity between amino acid sequences of the amplified chitinase III gene was 93.1% for all three Russian raspberry cultivars vs. unknown raspberry and 86.9% vs. strawberry. Twenty amino acid substitutions were the same for all three Russian and one unknown raspberry cultivars compared to strawberry sequence. In addition, eight substitutions were unique only for the unknown raspberry cultivar, and two substitutions were unique for our three cultivars.

## 4. Discussion

Modern plant breeding, including also berry crop breeding, seems to be almost impossible without modern genomic methods. They are needed to develop DNA based molecular genetic functional markers, such as single nucleotide polymorphisms (SNPs) and SSRs in adaptive and breeding trait related genes. The SSRs are highly variable in length due to insertion-deletion of the entire repeat units or motifs, mainly as a result of recombination errors or DNA polymerase slippage [34]. Genic SSRs could be even more informative than SNPs because unlike biallelic SNPs they usually have multiple alleles that can mark multiple alleles and haplotypes in these genes. It makes them very suitable for the MAS [35]. In comparison to random noncoding genomic SSRs the genic and EST-SSR markers are more transferable [21,36] and are better amplified [37] because of more conservative primer annealing sites. In addition, in silico development of genic and EST-SSR markers can now be relatively easily done using publicly available nucleotide sequence databases. Genic SSRs in functional genes can be used as “functional genetic markers”, and they have a much higher transferability across different taxa than random genomic SSRs [38]. Sargent et al. [39] used primer pairs based on the binding sites in the *Fragaria* exons flanking polymorphic introns and found that their transferability was significantly higher compared to the random genomic SSRs. However, genic SSRs are usually less polymorphic than random genomic SSRs, which can limit their use in MAS [34]. Thus, the most optimal marker could be those that have a SSR locus in a variable gene region such as intron and the primer annealing sites in the conserved exons flanking this intron. In case of long introns, it would be important to have at least one annealing site in an exon. However, information on the exon-intron structure is not available in the EST databases, more genome sequence data become available allowing to design reliable, consistent, polymorphic, functional, and transferable genic SSRs.

### 4.1. Choice of Genes and Genic SSR Marker Development

The flavonoid pathway is initiated by chalcone synthase (CHS) and involves more than 10 enzymes that act at early and late stages leading to the biosynthesis of different compounds such as flavonols, condensed tannins (proanthocyanidins) and anthocyanins (Figure 1). It is well known that pelargonidin-3-glucoside is a major anthocyanin in strawberry [40], whereas cyanidin-3-sophoroside is a major anthocyanin in red raspberry, followed by other cyanidin glycosides [41]. The late structural genes are regulated at transcriptional level by a ternary protein complex named MBW, which is formed by R2R3-MYB TFs, basic helix-loop-helix (bHLH) proteins, and WD40-repeat proteins [42].

For development of new SSR markers we used 13 structural and four regulatory flavonoid biosynthesis genes from GenBank (9 *F*. × *ananassa* and 8 *Rubus* species genes) (Table S1). Particular attention was paid to the flavonol synthase (*FLS*) and dihydroflavonol 4-reductase (*DFR*) genes, for which two SSR markers were developed. These enzymes competed for common substrates, dihydroflavonols (Figure 1), in order to direct the biosynthesis to colorless flavonols or colored anthocyanins, respectively, and may determine color phenotype [43]. For comparison, we also used a pair of primers designed for the *RiG001* locus from the *R. idaeus* aromatic polyketide synthase (*RiPKS3*) gene [28]. Unlike the RiPKS1, the typical naringenin chalcone synthase (CHS), the *RiPKS3* produced predominantly p-coumaryltriacetic acid lactone [44], but the sequences of both genes amplified by their PCR primer pairs are almost identical [23]. Among the *MYB TFs* genes we used the *MYB10* orthologs involved in the anthocyanin biosynthesis during ripening in more than 20 commercially important *Rosaceae* species [45]. Assuming its important role in flavonoid pathway regulation, we developed two markers for this gene. The *bHLH* gene from red raspberry is very similar to the *MdbHLH33* gene that is closely associated with anthocyanin production in apple [46]. In addition, we included in the study genes encoding PR proteins—chitinase (*FaChi2-1*) and ß-1,3-glucanases (*FaBG2-2* and *ToyoGluIII*). Previous studies demonstrated that expression of the *FaChi2-1* and *FaBG2-2* genes in strawberry are induced in response to pathogen inoculation [47].

A total of 118 SSR loci were identifies in 21 genes, and pentanucleotide motifs were the most abundant. Our result is in contrast to previous findings identifying trinucleotide [21,37] as the most frequent genic repeats in Rosaceae plant species, unlike dinucleotide motifs that were the most frequent among non-genic random genomic repeats [36,48,49]. The difference can be also explained, at least partly, by less stringent search parameters that were used in our study compared to those that are typically used in searches for random genomic SSRs, but they were similar with those that are usually used for the search of SSRs in coding regions. For example, Park et al. [37] found that the most profound allelic variation was revealed by the primer pairs flanking the penta-repeats (91%), whereas authors noted no significant difference among di-, tri-, and tetra-repeat (61–66%) motifs.

Identified SSRs were categorized by location in exons, introns, 5′UTRs, 3′UTRs, and upstreams. SSRs were located mainly in NCDS in genes: although exons occupied 45% of the gene length, but they contained only 28% of the SSR loci. It is known that genic SSRs located mainly in variable NCDS, but not in conserved exons. For example, development of genome-wide SSR markers in such different species as papaya and chickpea showed a similar distribution of SSRs across genome: 78–87% were in the intergenic regions, 9–10% in introns, and 2–3% in exons [34,50]. It has been repeatedly reported, that tri- and hexanucleotide motifs were more abundant in exons since they do not lead to frame shift and do not effect protein function and property as much as mutation of other repeats that could be under mutation pressure. Meanwhile, di-, tetra-, and pentanucleotide motifs are abundant in NCDS [35,36,50]. Such selection also reduces the variability of SSRs in exons. This is not consistent with our data, where pentanucleotide SSRs were the most common in the exons. However, apparently selection nevertheless occurred, and we did not find SSRs with mono-, di-, and tetranucleotide motifs in exons in our study, although they made up a significant part of all SSRs in introns and UTRs (Table 2). We found also that A and T nucleotides prevailed both in general in the studied genes (72%), and to a greater extent in NCDS (80%). It is known that some motifs, such as AT/TA, showed a greater abundance in most species [51].

When developing markers, we took into account the location of loci in genes (so that they were in different gene regions), as well as the location of several loci in the same marker. In addition, preference was given to dinucleotide motifs and a greater number of motif repeats, because such SSRs are more polymorphic [28,51]. As a result, we designed 15 and 9 primer pairs for SSR markers based on nucleotide sequences of genes with known function in *F*. × *ananassa* and *Rubus* species, respectively. These markers included all flavonoid biosynthesis genes available for *Fragaria* and *Rubus* in the NCBI GenBank database. Earlier, sets of SSRs for poplar genes involved in wood formation [52] or stress related genes in peanut [53] were developed, but not on flavonoid biosynthesis genes. We also developed SSR markers for the TF regulatory genes, which were not previously reported for *Fragaria* and *Rubus*.

### 4.2. Choice of Cultivars and Transferability of SSR Markers

It was shown that total anthocyanin content correlated with color of berry in both strawberry [54] and raspberry [55], and cultivars were selected primarily to have a broad variety of berry colors. In addition, we took into account their commercial value and application in the breeding programs. Other *Rubus* and *Fragaria* cultivars were chosen due to their diverse ploidy (black raspberry, blackberry, loganberry, and boysenberry) and as wild potential donors of traits of interest (*F. vesca* and *F. viridis*). The rare cultivated species of *Rubus* were also included in the study, such as cloudberry (*R. chamaemorus*), arctic bramble (*R. arcticus*), and hybrid arctic bramble (*R. × stellarcticus*). Only a few rare reports on the SSR markers are available for these boreal species [56,57] that are rich in ellagic acid and are regionally extremely important and valuable crops. Moreover, *R. arcticus* is used to develop new cultivars. The hybrids of the octoploid *F*. × *ananassa* and the hexaploid *C. palustre*, which unlike the white-flowered strawberry have red and pink flowers and are usually grown for ornamental purposes, are also of high interest for the flavonoids biosynthesis research.

The amplification of the SSR markers in the strawberry specimens using primer pairs based on the *F*. × *ananassa* sequences was 100% successful, and this is in agreement with data obtained by many other researchers. The transferability of the strawberry SSR markers to *F. vesca* and *F. viridis* was noticeably lower (73.3%): *FaMY01*, *FaMY02*, *FaDR01*, and *FaLR01* were not amplified using primer pairs developed from *F*. × *ananassa* genes. It is known that rapid genomic changes can occur after polyploidization, and loss of homologous copies of many duplicated genes was often observed [21]. Hirakawa et al. [58] calculated that the size of the octoploid *Fragaria* genome (698 Mb) approximately equalled 80% of the combined genomes of its four diploid wild relatives, *F. iinumae*, *F. nipponica*, *F. nubicola*, and *F. orientalis* (~200 Mb each). It is also possible that primer binding sites in *F. vesca* and *F. viridis* for these SSR markers have mismatches with sites that were used to design primers. It cannot be also excluded that the prolonged selection (with the purpose to change the quality of strawberries) contributed to the discrepancy between binding sites. The alleles unique for some diploid species were identified at a number of loci. The marker transferability to (*F*. × *ananassa*) × *C. palustre* hybrid is not different from the strawberry specimens. This happened due to the fact that *C. palustre* is a close relative of *Fragaria*—they are members of the same subtribe Fragariinae [1]. Twelve out 15 markers (80%) were polymorphic in *F*. × *ananassa* (produced two and more fragments). This is consistent with other authors who reported 81% [59] and 91% [21] of polymorphic loci.

The transference among the *Rubus* species was not as successful as among *Fragaria* species. The transferability of loci from the *Rubus* flavonoid pathway genes was 70% for red raspberry, 50% for hybrid arctic bramble, and 60% for the other *Rubus* species. This fact suggests a close genetic relationship among the studied *Rubus* species and a high conservativeness of the flavonoid biosynthesis genes. Mnejja et al. [16] showed that transferability negatively correlated with genetic distance between the genera in the Rosaceae family and between species within the *Prunus* genus. However, many authors reported similar and high (80–100%) cross-species amplification of raspberry loci (*Idaeobatus* subgenus) in other species, such as black raspberry (*Idaeobatus* subgenus) [60,61], blackberry (*Rubus* subgenus) [28,60,61], and Arctic bramble (*Cyclastis* subgenus) [57]. The *RiG001* marker was amplified only in red raspberry. Castillo et al. [28] found that this marker was not amplified in blackberry and hybrids. We demonstrated it also in black raspberry earlier [23] and in cloudberry, arctic bramble, and hybrid Arctic bramble in this study. This marker appears to be a good identifier for red raspberry among cultivated *Rubus* species.

The transferability of *F*. × *ananassa* loci to *Rubus* species was moderate: 46.7% in Nordic species, 40% in red raspberry and hybrids, and 33.3% in black raspberry and blackberry (33.3%). Other authors [20,62] reported even a lower transferability from *F*. × *ananassa* to diploid red and black raspberries (*Idaeobatus* subgenus, 8–23%) than to tetraploid blackberry (*Rubus* subgenus, 26–36%). Only two of the 15 strawberry markers were polymorphic in raspberries (*FaFS01* and *FaCH01*) and five markers if all *Rubus* species were taken into account. This is consistent with other data that demonstrated the difficulty of identifying polymorphic loci in *Rubus*—only 10% of the markers from *Fragaria* amplified a polymorphic product in *Rubus* [17].

There are no published data on transferability of markers from *Rubus* to *Fragaria*. In our study, only three of 10 *Rubus* primer pairs amplified in all *Fragaria* specimens. All of them were from *R. idaeus* (the *ANS* locus and two TF genes—*MYB10* and *bHLH*). This level is slightly higher than the transferability from *Fragaria* to *Rubus*. It should be noted that the primers for the similar genes in *Fragaria*, *ANS* and *MYB10*, did not amplified in *Rubus*. The amplification of *RiMY01* showed the presence of unique alleles in *F. vesca* and *F. viridis*.

The moderate transferability level between *Fragaria* and *Rubus* suggests a remote relationship between these two genera. Potter et al. [1] reported that the phylogenetic relationship between *Fragaria* and *Rosa* is closer than between *Fragaria* and *Rubus*. Qi et al. [63] evaluated the SSR primer pairs using in silico PCR and demonstrated that strawberry is the closest to the rose followed by the raspberry. We found an interesting regularity in the size of alleles during cross-species amplification: alleles in *Rubus* species amplified using *Fragaria* primers had sizes similar to the expected, while alleles in *Fragaria* species amplified using *Rubus* primers were significantly smaller than expected. This was not previously reported, since there was no work on the transferability of *Rubus* markers to *Fragaria*. Perhaps this is due to the fact that the genus *Rubus* is evolutionary older than the *Fragaria* genus [1,64].

The SSR transferability to the poorly studied northern *Rubus* species did not differ from raspberries and blackberries, except for the amplification of the *FaLR01* marker in them. Kostamo et al. [57] reported amplification of all seven markers in arctic bramble cultivars using primer pairs developed for raspberry SSR loci, but the annealing temperature was lowered to 50 °C from the original 60 °C. It is known that lowering the annealing temperature may increase transferability, but nonspecific amplification can occur [21]. We have repeatedly observed null-alleles among hybrid arctic bramble cultivars for the *FaFS01*, *RcFH01*, *FaLR01*, *FaFH01*, and *FaCH01* markers and only once for cultivars of other *Rubus* species (for FaCH01 marker). This is probably due to the high heterozygosity in primer binding sites that results in mismatch between primers and binding sites in the *R.* × *stellarcticus* cultivars. Closer similarity of allele sizes to strawberry markers in northern *Rubus* species compared to raspberries and blackberries, as well as the amplification of the *FaLR01* marker in them, suggests their closer relationship with *Fragaria* compared to other tested *Rubus* species.

### 4.3. Genetic Diversity of Fragaria and Rubus

The number of alleles varied among 12 SSR markers genotyped in 13 strawberry specimens and ranged from two to 14 alleles (6.67 alleles on average) (Table 3). This is slightly higher than previously reported for *F*. × *ananassa* (5.6) [65]. The low number of alleles per locus can reflects a poor choice of microsatellites or low levels of genetic diversity. Hilmarsson et al. [10] found a mean number of alleles in *F. vesca* was only 4.5 and the authors believed that this was due to low levels of genetic diversity in the species, but the most polymorphic marker had 16 alleles. Within the strawberry collection, the mean *H_o_* and *H_e_* were 0.63 and 0.66, respectively, and the mean polymorphism information content (PIC) was 0.66. Our *H_e_* value is similar to 0.66 obtained by Yoon et al. [65], but *H_o_* and PIC significantly exceed their values of 0.51 and 0.45, respectively. The mean *H_o_* and *H_e_* vary significantly across different *Fragaria* species: from 0.08 and 0.17 in *F. vesca* [10] up to 0.75 and 0.86 in *F. virginiana* [66], respectively. Nine informative markers having high polymorphism information content (PIC) values (0.52–0.89) can be used for efficient evaluation of large collections of strawberry samples.

The parameters of polymorphism were also calculated for 24 diploid *Rubus* cultivars based on 10 SSR markers (Table 4). From two to 13 (mean 4.6) alleles were observed for polymorphic markers in diploid *Rubus* cultivars. These values were rather similar with published results obtained in other diploid *Rubus* species. From two to 5 (mean 3) alleles per polymorphic locus were observed in 21 *R. occidentalis* cultivars [67] and 2–15 (7.5) in 24 *R. idaeus* cultivars [28]. In our study, the mean values of *H_o_*, *H_e_*, and PIC were 0.23, 0.51, and 0.46, respectively. These values were lower than those observed in *R. idaeus* [28] and *R. coreanus* [49]. This may be because the gene sequences, from which our SSR markers are derived, are more conserved compared to random genomic SSRs used in these published reports. However, average *H_e_* and PIC in our study were higher than in the published study in *R. occidentalis* [67], while *H_o_* was lower. The most polymorphic SSR loci based on high *H_o_*, *H_e_*, and PIC were *RiMY01* and *FaFS01*. *FaFS01* was also the most polymorphic marker for strawberry. These loci contained long dinucleotide repeats, which usually have higher levels of polymorphism compared to other repeats [21]. The *RiG001* marker demonstrated the PIC value, that was very similar with results, reported by Castillo et al. [28]—0.43 vs. 0.46, respectively. Our results demonstrated that the SSR markers developed in this study might be useful for the genetic assessment of *Fragaria* and *Rubus* species.

PCA of 16 *Fragaria* specimens genotyped with 13 SSR markers demonstrated clear separation between *F*. × *ananassa*, *F. vesca*, *F. viridis*, and *Fragaria* × *Comarum* hybrid (Figure 2). Biswas et al. [68] also demonstrated a clear separation of 26 *F*. × *ananassa* and 7 *F. vesca* specimens by SSR. Vallarino et al. [69] showed a clear separation between domesticated (*F*. × *ananassa*) and wild (*F. moschata*, *F. vesca*, and *F. chiloensis*) specimens using profiles of primary and secondary metabolites, but wild species formed a common cluster. Unlike our study, Sanchez-Sevilla et al. [70] showed no differences between (*F*. × *ananassa*) × *C. palustre* hybrid (Pink Panda cultivar) and strawberry cultivars using РСА based on DArT markers, although hybrid and cultivars were most diverse according to the phylogenetic dendrogram.

PCA of 32 *Rubus* cultivars genotyped with 10 SSR markers demonstrated, in general, a good separation of the four *Rubus* subgenera, except *R. occidentalis*, which clustered separately from *R. idaeus* despite belonging to the same *Idaeobatus* subgenus (Figure 3). Earlier, PCA of 50 *Rubus* cultivars based on genomic SSRs showed that black raspberry is the most distant from other *Rubus* species [60]. Interestingly, in report of Simlat et al. [71], Jewel cultivar was not separated from red raspberry using PCA based on SSRs. According to our data, *Rubus* hybrids clustered together with red raspberry, while in Graham et al. [60] they were located approximately in the middle between red raspberry and blackberry cultivars.

### 4.4. Relationship between Properties of SSR Markers and Their Location

The more transferable SSR markers are characterized by a lower frequency of null alleles. The most likely reason for null alleles are mutations in one or two primer binding sites creating mismatch between primer and binding site sequences [22]. The chances for mismatch depend on location of these sites in gene, and, therefore, location of binding sites is important for amplification. We showed a clear relationship between the location of primer binding sites and transferability of SSR markers within and between *Fragaria* and *Rubus* genera (Table 5). The most transferability demonstrated markers that were amplified by primer with binding sites located in exons. The location of binding sites in NCDS resulted in a significant decrease of transferability. It should be noted that location only one of two binding sites in variable sequences was sufficient to drastically reduce transferability, especially between genera. It is known, that chances of mutations in binding sites and their mismatch with primers will increase in taxa that are phylogenetically distant [48]. In our study, all null alleles in some *R. × stellarcticus* hybrids were observed only for loci with primer binding sites located in NCDS.

The lowest transferability was found also when one of the two primer binding sites were located across the intron/exon junction. Three out of four markers with such binding sites were not amplified in any of the 48 tested specimens, even in the blackberry which sequences were used to develop these markers. Thus, we experimentally confirmed the assumption of Vidal et al. [34] that PCR amplification failures can occur in the case of designing primers with binding sites across exon-intron junctions. It should be noted that for two SSR loci detected in the *RhDFR* gene and one SSR in the *RhANR* gene all computer programs used in this study to design primers selected the intron-exon junctions for binding sites, and these two markers were not amplified. Thus, either parameters for designing primers for these markers should be changed or primers should be designed manually to develop successful SSR markers for these genes.

The polymorphism of SSR markers in transcribed regions can affect transcription, translation, and/or gene function. SSR polymorphisms within exons can result in amino acid change that can lead to a gain or loss of function, in the 5′UTR they can regulate gene expression by affecting transcription and translation, in the 3′UTR they can be responsible for gene silencing or transcription slippage, and in introns they can affect gene transcription, mRNA splicing, or export to cytoplasm [35,52]. Ultimately, all these polymorphisms can affect phenotypes. However, the likelihood of polymorphism in different gene regions may vary. Our studies have shown a clear association between polymorphism of SSRs and their location in CDS and NCDS (Table 5). Highly polymorphic loci were located in introns, 5′UTRs, and upstreams, whereas loci with moderate polymorphism and monomorphic ones were located in exons. This relationship has been observed for both *Fragaria* and *Rubus*. For example, the *FaFS01* marker representing SSR in the second intron of the flavonol synthase gene was one of the most polymorphic in strawberries (14 alleles) and *Rubus* (eight alleles), while the *FaFS02* marker representing SSR in the first exon of the same gene was monomorphic in both genera. In general, SSRs located in introns had 5.5 and 6.0 alleles per locus on average in *Fragaria* and *Rubus*, respectively, whereas in SSRs located in exons—only 1.8 and 3.0 alleles per locus, respectively. Our results confirm the data of Du et al. [52] that intronic SSRs of *Populus tomentosa* were more variable (3.7 alleles/locus) than exonic SSRs (2.4 alleles/locus), likely due to higher selection pressure on CDS than on NCDS.

### 4.5. SSR Markers Representing Transcription Factor (TF) Genes

The TFs of MYB and bHLH families are widespread in plants. Using the lily transcriptome Biswas et al. [72] developed 71 SSRs in genes of 31 different TF families and most of them represented the bHLH TF (10 SSRs) and MYB (nine SSRs) families. The SSR markers in the TF gene in plants were first developed by Kujur et al. [73], which showed the influence of the SSR polymorphism on secondary structure of proteins and on such traits as seed weight and number of pods and seeds. Since then, there have been few similar studies in Rosaceae where similar markers have been developed for Japanese plum [24], but no such markers have been reported for *Rubus* and *Fragaria* before our first study, where we developed a SSR marker representing the *RiMYB10* TF gene, an activator of transcription of flavonoid biosynthesis [23]. The association between the *MYB10* gene and fruit color has been demonstrated for apple [45] and peach [74]. Gonzalez et al. [24] found three allele variants in the EST–SSRs designed for the *PsMYB10* TF gene. In our study of 21 varieties of red and black raspberries presented here, we showed that the *RiMY01* marker representing the *R. idaeus MYB10* gene had a significantly greater polymorphism compared to six markers representing structural genes of flavonoid biosynthesis—9 vs. 2–4 alleles per locus and *PIC* of 0.82 vs. 0.05–0.35 [23]. This is consistent with high PCR amplification efficiency and high polymorphism found for SSR markers representing TF genes in a number of crops [72].

In this study, we developed five SSRs using sequences of all publicly available TF genes of *Rubus* and *Fragaria*, which are part of the MBW transcriptional complex. As a result, two of the three most multiallelic markers were the *RiMY01* and *FaMY02* from TF genes. We assume that their high variability is caused by a combination of several factors: arrangement in introns, long dinucleotide repeats, and a noticeable AT enrichment. It is interesting that according to Biswas et al. [72] the SSRs in most TF genes were GC-rich, but genes in the bHLH and MYB families have higher AT content than other GC-rich genes. It is possible that this is a feature of these TF families, causing their increased variability. High level polymorphism of these SSR markers suggests association of alleles in flavonoid biosynthesis TF genes with variation in flavonoid accumulation and composition. Thus, we believe that SSR markers developed in this study and representing the TF genes can serve as useful tools for MAS of *Fragaria* and *Rubus* species.

### 4.6. Chitinase III as Allergen in Raspberry

Chitinases are able to degrade the chitin, a major component of fungal cell walls, and they play key roles in plant defense system from fungal pathogens. Plant chitinases are also a well-known group of food allergens. It is a relatively small group, but it is present in highly consumed fruits [75]. Chitinases can be divided into five classes (I-V), and plant class III chitinases (PR-8 proteins) have an additional lysozyme activity, which is not found in other classes of chitinases [76]. Marzban et al. [33] identified four allergen proteins including class III chitinase in raspberry fruits. They showed high sequence identity of raspberry proteins to different PR protein families in Rosaceous species (especially to Fra a strawberry proteins) and suggest that the consumption of raspberries might be responsible for adverse reactions in sensibilized individuals. We sequenced fragments of chitinase III genes in three Russian raspberry cultivars that have yellow-, orange- and red-colored berries and compared them with the chitinase III genes in Chandler strawberry (GenBank AF134347) and unknown raspberry [33]. These sequences matched both raspberry and strawberry sequences, but there were nonsynonymous substitutions in the raspberry sequences. Two nonsynonymous substitutions differed both strawberry and previous published raspberry chitinase III sequences. The amino acid sequences of all three raspberry cultivars with different berry colors were identical.

It is known that fruit color correlates with allergen content in strawberry. Hjerno et al. [77] demonstrated that the strawberry Fra a 1 allergen (a homolog of the major birch pollen allergen Bet v 1) is synthesized in red ripe fruits of *F.* × *ananassa*, but not in white (colorless) cultivars. Proteomic analyses have shown that Fra a 1 allergen and several enzymes of the flavonoid biosynthesis pathway were down-regulated. Later, suppression of three Fra a genes using RNAi approach alters phenolic compound levels in strawberry and led to decreased accumulation of main anthocyanins responsible for the red color of fruits [78]. Finally, Casanal et al. [79] suggested that Fra a proteins may play an important role in the control of flavonoid pathways by binding to metabolic intermediates. It can be assumed that a similar connection exists in raspberry, and that future studies in this direction are necessary. If this hypothesis is confirmed, yellow-colored raspberry cultivars will have less allergenic potential and are suitable for feeding children with increased allergenic sensitivity.

## 5. Conclusions

In this study, we developed a set of SSR markers representing structural and regulatory flavonoid biosynthesis genes of berry crops. These genic SSRs have been successful to identify allelic variations in *Fragaria* and *Rubus* species with contrasting color berry phenotypes. The results suggest that boreal *Rubus* species are more related to *Fragaria* compared to raspberry and blackberry. We demonstrated a clear relationship between transferability of SSR markers within and between the *Fragaria* and *Rubus* genera and location of primer binding sites and between the marker polymorphism and its location in coding and non-coding sequences. The SSR markers representing TF genes showed high allelic variability and may be good candidates for MAS in berry species. The genic SSRs developed in this study may be used for future genetic diversity and population genetics studies in *Fragaria* and *Rubus* species, as well as may be considered as candidate markers in breeding programs for improvement anthocyanin related traits.

## Figures and Tables

**Table 1:** List of 48 *Fragaria* and *Rubus* specimens genotyped in the study.

Species	Specimen	Pedigree	Ploidy	Origin
*F*. × *ananassa*	White D	*F. virginiana* × *F. chiloensis*	8*x*	Sweden
	Vechnaya Vesna	Grenader-V2 × Rannyaya Plotnaya	8*x*	Russia
	Girlyanda	Elsanta × Korona	8*x*	Russia
	Zolushka	Festivalnaya × Senga Sengana	8*x*	Russia
	Lakomka	Krasavitsa × Korona	8*x*	Russia
	Lyubava	Solovushka × Geneva	8*x*	Russia
	Solovushka	Syurpriz Olimpiade × Festivalnaya Romashka	8*x*	Russia
	Tsaritsa	Venta × Red Gauntlet	8*x*	Russia
	Honeoye	Vibrant × Holiday	8*x*	USA
	Korona	Tamella × Induka	8*x*	Netherlands
	Red Gauntlet	New Jersey 105 × Climax	8*x*	UK
	Senga Sengana	Sieger × Markee	8*x*	Germany
	Black Prince	unknown	8*x*	unknown
*F*. × *Comarum*	Lipstick	Hybrid (*F*. × *ananassa*) × *Comarum palustre*	7*x*	Netherlands
*F. vesca*	-	wild strain	2*x*	Russia
*F. viridis*	-		2*x*	Russia
*R. chamaemorus*	NyBy		8*x*	Finland
*R. arcticus*	Elpee		2*x*	Finland
	Mespi		2*x*	Finland
*R. × stellarcticus*	Anna	Hybrid arctic bramble *R. arcticus* ssp. *stellatus* × *R. arcticus* ssp. *arcticus*	2*x*	Sweden
	Beata		2*x*	Sweden
	Linda		2*x*	Sweden
	Sofia		2*x*	Sweden
	Astra		2*x*	Finland
	Aura		2*x*	Finland
*R. idaeus*	Babye Leto II	Autumn Bliss × Babye Leto	2*x*	Russia
	Oranzhevoe Chudo	Shapka Monomaha (open pollination)	2*x*	Russia
	Zheltyj Gigant	Marosejka × Ivanovskaya	2*x*	Russia
	Zolotaya Osen	13-39-11 (open pollination)	2*x*	Russia
	Patritsiya	Marosejka × М102	2*x*	Russia
	Gusar	Canby × pollen mix	2*x*	Russia
	Fenomen	Stolichnaya × Odarka	2*x*	Ukraine
	Joan J	Terri-Louise × Joan Squire	2*x*	UK
	Marosejka	7324/50 × 7331/3	2*x*	Russia
	Pingvin	interspecific hybrid	2*x*	Russia
	Fall Gold	NH 56-1 × (Taylor × *R. pungens* var. oldhamii) F2 (open pollination)	2*x*	USA
	Himbo Top	Autumn Bliss × Rafzeter	2*x*	Switzerland
	Polana	Heritage × Zeva Herbsterne	2*x*	Poland
	Zhar-Ptitsa	7-43-2 (open pollination)	2*x*	Russia
*R. occidentalis*	Cumberland	Gregg selfed	2*x*	USA
	Jewel	(Bristol × Dundee) × Dundee	2*x*	USA
Blackberry	Brzezina	90,402 × 89,403	4*x*	Poland
	Natchez	Ark. 2005 × Ark. 1857	4*x*	USA
	Ebony King	unknown	4*x*	USA
Hybrid	Boysenberry	complex hybrid	7*x*	USA
	Loganberry	*R. ursinus* × *R. idaeus*	6*x*	USA
	Tayberry	Aurora × SCRI 626/67	6*x*	UK
	Buckingham Tayberry	chimeral spineless sport of Tayberry	6*x*	UK

**Table 2:** Number of microsatellite loci with different nucleotide repeat motifs and their location in gene.

Location	SSR Motif						Total
	Mono	Di	Tri	Tetra	Penta	Hexa	
Upstream	0	2	2	1	5	7	17
5′UTR	0	3	0	2	7	4	16
Exon	0	1	3	0	18	11	33
Intron	2	9	0	6	17	7	41
3′UTR	4	0	0	0	6	1	11
Total	6	15	5	9	53	30	118

**Table 3:** Diversity of 12 polymorphic SSR markers in 13 *F*. × *ananassa* specimens.

Locus	Location	Number of Alleles	*H_o_*	*H_e_*	PIC
*FaF3H01*	5′UTR	4	0.42	0.52	0.52
*FaFH01*	intron	5	0.78	0.72	0.79
*FaFS01*	intron/exon	14	0.90	0.90	0.89
*FaDR01*	upstream	9	0.58	0.82	0.81
*FaLR01*	intron	4	0.77	0.71	0.70
*FaFG01*	5′UTR/exon	8	0.74	0.72	0.71
*FaMY01*	intron	4	0.42	0.45	0.45
*FaMY02*	intron	13	0.75	0.84	0.83
*RiMY01*	intron	2	0.48	0.50	0.50
*RiHL01*	intron	4	0.60	0.60	0.59
*FaCH02*	exon	4	0.37	0.35	0.34
*FaBG01*	upstream	9	0.68	0.82	0.81
Mean		6.7	0.63	0.66	0.66

**Table 4:** Diversity of 10 polymorphic SSR markers in 24 diploid *Rubus* cultivars.

Marker	Location	Number of Alleles	*H_o_*	*H_e_*	PIC
*RiG001*	intron	3	0.08	0.53	0.43
*FaFH01*	intron	5	0.00	0.57	0.51
*RcFH01*	intron	4	0.21	0.43	0.40
*FaFS01*	intron/exon	5	0.29	0.70	0.65
*FaLR01*	intron	3	0.04	0.34	0.29
*RiAS01*	exon	4	0.33	0.62	0.56
*RhUF01*	exon	4	0.33	0.38	0.34
*RiMY01*	intron	13	0.42	0.84	0.82
*RiHL01*	intron	2	0.04	0.04	0.04
*FaCH01*	5′UTR/exon	3	0.50	0.64	0.57
Mean		4.60	0.23	0.51	0.46

**Table 5:** Relationships between transferability and polymorphism of SSR markers and location of primer pairs and SSR loci in gene.

Genus	Locus	Primer Binding Site Location ^1^	Amplification		Locus Location	*F*. × *ananassa*		*Rubus* ^3^	
			*Fragaria*	*Rubus* ^2^		Number of Alleles	Polymorphism	Number of Alleles	Polymorphism
**Both Binding Sites Located in Exons**									
*Fragaria*	*FaFH01*	ex1–ex2	+	+	in1	5	+	8	–/+/+/+/–/+
	*FaFS01*	ex2–ex3	+	+	in2 ex3	14	+	8	+/–/+/+/–/+
	*FaFS02*	ex1–ex1	+	+	ex1	1	–	1	–
	*FaAR01*	ex4–ex5	+	+	ex4 in4	2	–	1	–
	*FaTG01*	ex2–ex2	+	+	ex2	1	–	3	–/–/–/+/–/–
*Rubus*	*RiAS01*	ex2–ex2	+	+	ex2	2	–	5	+/–/+/+/+/+
	*RhUF01*	ex2–ex2	–	+	ex2	–	n	5	+
	*RiTT01*	ex–ex	–	+	exon	–	n	1	–
**One of Binding Sites Located in Exon another in Intron or 5′UTR**									
*Fragaria*	*FaMY02*	in2–ex3	+/–	–	in2	14	+	–	n
	*FaLR01*	ex2–in2	+/–	–/–/–/+/+	in2	4	+	2	n/n/n/n/+/+
	*FaFG01*	5′UTR–ex1	+	–	5′UTR ex1	8	+	–	n
	*FaAS01*	in–ex2	+	–	ex2	1	–	–	n
	*FaCH01*	5′UTR–ex1	+	+/–/+/+/+	5′UTR ex1	2	–	3	+/n/n/+/–/+
	*FaCH02*	in–ex2	+	–	ex2	4	+	–	n
*Rubus*	*RiHL01*	ex1–in	+	+	in	4	+	2	+/–/–/–/–/–
	*RcFH01*	in2–ex3	–	+	in2	–	n	5	+/+/–/+/–/+
	*RiG001*	in–ex2	–	+/–/–/–/–	in	–	n	2	+/n/n/n/n/n
**Both Binding Sites Located in Intron, 5′UTR or Upstream**									
*Fragaria*	*FaMY01*	in2–in2	+/–	–	in2	4	+	–	n
	*FaDR01*	up–5′UTR	+/–	–	up	9	+	–	n
	*FaF3H01*	5′UTR–5′UTR	+	–	5′UTR	4	+	–	n
	*FaBG01*	up–up	+	–	up	9	+	–	n
**One of Binding Sites Located across Exon/Intron Junction**									
*Rubus*	*RiMY01*	ex1/in1–ex2	+	+/+/+/+/–	in1	2	+	17	+/–/+/+/+/n
	*RhDR01*	in2–in2/ex3	–	–	in2	–	n	–	n
	*RhDR02*	ex1–in1/ex2	–	–	in1	–	n	–	n
	*RhAR01*	ex5–ex5/in5	–	–	e5	–	n	–	n

^1^ in—intron, ex—exon; up—upstream; the number is not provided in case, if gene has only a single or no intron. ^2^ *R. idaeus*/*R. occidentalis* and blackberry/hybrids/*R. chamaemorus* and *R. arcticus*/*R. × stellarcticus.* ^3^ *R. idaeus*/*R. occidentalis*/blackberry/hybrids/*R. arcticus*/*R. × stellarcticus.* n—no amplification.

## Supplementary Materials

The following are available online at https://www.mdpi.com/2073-4425/11/1/11/s1, Figure S1: Alignment of the chitinase III amino acid sequences from strawberry and raspberry cultivars *F*. × *ananassa* cv. Chandler (AF134347), *R. idaeus* of unknown origin (Marzban et al. [33]), *R. idaeus* cvs. Zolotaya Osen (MK333194), Oranzhevoe Chudo (MK333195), and Babye Leto II (MK333196). Identical amino acid positions are highlighted by the same color, Table S1: Data on 25 SSR loci and their PCR primer pairs used to genotype *Fragaria* and *Rubus* specimens.
