# Supplementary material for: Transferability and Polymorphism of SSR Markers Located in Flavonoid Pathway Genes in Fragaria and Rubus Species
Source: Genes (Basel). 2019 Dec 21;11(1):11. doi: 10.3390/genes11010011 (PMC7017068; doi:10.3390/genes11010011)
Supplement: Supplementary file 1 [file genes-11-00011-s001.zip › FigS1_final.pdf]

```
Reference sequence (1): F._x_ananassa_(cv_Chandler)
Identities normalised by aligned length.
Colored by: identity

                                     cov    pid   1 [         .         .         .         .         :         . 60
1 F._x_ananassa_(cv_Chandler)     100.0% 100.0%     MASVSAAALLSLATLLLLAVGSNAGGIAIYWGQNGNEGTLAQTCASGNYQFVNIAFHSSF
2 R.idaeus_(unknown)               90.0%  83.6%     ------------------------------WGQNGNEGTLXQTCASGNYQFVNIAFLSSF
3 R.idaeus_(cv_Zolotaya_Osen)      58.9%  86.9%     ------------------------------------------------------------
4 R.idaeus_(cv_Oranzhevoe_Chudo)   58.9%  86.9%     ------------------------------------------------------------
5 R.idaeus_(cv_Babye_Leto_II)      58.9%  86.9%     ------------------------------------------------------------

                                     cov    pid  61           .         .         .         1         .         . 120
1 F._x_ananassa_(cv_Chandler)     100.0% 100.0%     GNGRTPTLNLAGHCDPSSNTCTKFSSQIKSCQAEGIKVILSVGGGWGGQYSLASSEDARQ
2 R.idaeus_(unknown)               90.0%  83.6%     GNGQTPTINLAGHCDPSTNECTKLSPDIKSCQAEGIKVILSIGGGAGGSYSLASPNDARQ
3 R.idaeus_(cv_Zolotaya_Osen)      58.9%  86.9%     ---------------------------------------------------------ARQ
4 R.idaeus_(cv_Oranzhevoe_Chudo)   58.9%  86.9%     ---------------------------------------------------------ARQ
5 R.idaeus_(cv_Babye_Leto_II)      58.9%  86.9%     ---------------------------------------------------------ARQ

                                     cov    pid 121           .         .         :         .         .         . 180
1 F._x_ananassa_(cv_Chandler)     100.0% 100.0%     VGAYLWNNFLGGHSTSRPLGDAVLDGVDFDIEGGNDQYWDDLARYLSAHSKKGGKKVYLT
2 R.idaeus_(unknown)               90.0%  83.6%     VATYLWNNFLGGQSSSRPLGDAVLDGVDFDIEGGTDQHWDDLARYLSGYSKKGGKKVYLT
3 R.idaeus_(cv_Zolotaya_Osen)      58.9%  86.9%     VGTYLWNNFLGGQSSSRPLGDAVLDGVDFDIEGGTDQYWDDLARYLSAHSNKGGKTVYLT
4 R.idaeus_(cv_Oranzhevoe_Chudo)   58.9%  86.9%     VGTYLWNNFLGGQSSSRPLGDAVLDGVDFDIEGGTDQYWDDLARYLSAHSNKGGKTVYLT
5 R.idaeus_(cv_Babye_Leto_II)      58.9%  86.9%     VGTYLWNNFLGGQSSSRPLGDAVLDGVDFDIEGGTDQYWDDLARYLSAHSNKGGKTVYLT

                                     cov    pid 181           .         2         .         .         .         . 240
1 F._x_ananassa_(cv_Chandler)     100.0% 100.0%     AAPQCPFPDANIGNALKTGLFDNVWVQFYNNPPCQYTSGNVTNLEDAWKQWTSAIPAQQV
2 R.idaeus_(unknown)               90.0%  83.6%     AAPQCPFPDAWVGNALNTGLFDYVWVQFYNNPPCQYTSGDISNLEDAWKQWTSAIPTHKI
3 R.idaeus_(cv_Zolotaya_Osen)      58.9%  86.9%     AAPQCPFPDAWVGKALKTGLFDYVWVQFYNNPPCQYTSGDVTNLEDAWKQWTSAIPAHKI
4 R.idaeus_(cv_Oranzhevoe_Chudo)   58.9%  86.9%     AAPQCPFPDAWVGKALKTGLFDYVWVQFYNNPPCQYTSGDVTNLEDAWKQWTSAIPAHKI
5 R.idaeus_(cv_Babye_Leto_II)      58.9%  86.9%     AAPQCPFPDAWVGKALKTGLFDYVWVQFYNNPPCQYTSGDVTNLEDAWKQWTSAIPAHKI

                                     cov    pid 241           :         .         .         .         .        ] 299
1 F._x_ananassa_(cv_Chandler)     100.0% 100.0%     FLGLPAAPEAAGSGFIPAAALTTTVLPGIKTSDKYGGVMLWSKYYDDLYGYSSSIKNHV
2 R.idaeus_(unknown)               90.0%  83.6%     FLGLPAAPQAAGSGFIPAADLNSQVLPAIKNSAKYGGVMLWSKYYDDLDGYSASIKNDV
3 R.idaeus_(cv_Zolotaya_Osen)      58.9%  86.9%     FLGLPAAPQAAGSGFIPAADLNSQVLPAIKNSAKYGGVMLWSKYYDDLDGYSS------
4 R.idaeus_(cv_Oranzhevoe_Chudo)   58.9%  86.9%     FLGLPAAPQAAGSGFIPAADLNSQVLPAIKNSAKYGGVMLWSKYYDDLDGYSS------
5 R.idaeus_(cv_Babye_Leto_II)      58.9%  86.9%     FLGLPAAPQAAGSGFIPAADLNSQVLPAIKNSAKYGGVMLWSKYYDDLDGYSS------
```

Figure S1. Alignment of the chitinase III amino acid sequences from strawberry *Fragaria × ananassa* cv. Chandler (AF134347) and raspberry *Rubus idaeus* cultivars Zolotaya Osen (MK333194), Oranzhevoe Chudo (MK333195), Babye Leto II (MK333196), and of unknown origin (Marzban et al. 2008 [33]). Identical amino acid positions are highlighted by the same color.
