# Supplementary material for: Transferability and Polymorphism of SSR Markers Located in Flavonoid Pathway Genes in Fragaria and Rubus Species
Source: Genes (Basel). 2019 Dec 21;11(1):11. doi: 10.3390/genes11010011 (PMC7017068; doi:10.3390/genes11010011)
Supplement: Supplementary file 1 [file genes-11-00011-s001.zip › TableS1.pdf]

Table S1. Data on 25 SSR loci and their PCR primer pairs used to genotype *Fragaria* and *Rubus* specimens.

Locus	Gene	GenBank accession number	Motif and number of repeats	SSR location	Primer binding site location[1]	Forward (F) and reverse (R) primer nucleotide sequencies	T, Cº	Allele size, bp		
								expected	in *Fragaria*	in *Rubus*
RiG001	aromatic polyketide synthase (PKS3)	AF292369	$(AT)_6$	in	in–ex2	F: TGTCCGATCCTTTTCTTTGG<br>R: CGCTTCTTGATCCTTGACTTGT	55	345	-	0, 349, 351
FaF3H01	flavonoid 3'-hydroxylase	KC708489	$(AT)_8$<br>$(AACAA)_2$<br>$(TTCTT)_2$	5′UTR	5′UTR-5′UTR	F: TCCACATCTCTCTCCCATTCAT<br>R: GGTGTGCCGAAGAGTTAGTAGG	60	359	300-357	-
FaFH01	flavanone 3-hydroxylase	AY691919	$(ATTTT)_2$	in1	ex1–ex2	F: TCATCGTTTCCAGCCATTTAC<br>R: ATGCCTCCTTCTCTAAACCCAT	60	376	346-378	0, 341-372
RcFH01	flavanone-3-hydroxylase	EU255776	$(TATG)_3$	in2	in2–ex3	F: GGTCCAAGTGCATTCCATATTAC<br>R: GTTCTTGAATCTCCCGTTGCT	60	262	-	0, 255-299
FaFS01	flavonol synthase	DQ834905	$(CT)_{12}$-$(CA)_{12}$<br>$(AAGAC)_2$	in2<br>ex3	ex2–ex3	F: CATCCCTAATGCCCTAGTCATC<br>R: TGTACTTCGGTGGATTCTCCTT	60	304	272-315	0, 291-336
FaFS02	flavonol synthase	DQ834905	$(GGAAG)_2$	ex1	ex1–ex1	F: AAGCTCCTCAAACAAATCTTCG<br>R: GTAGTTAATGGCAGAAGGTGGC	60	273	253, 271	271
FaDR01	dihydroflavonol 4-reductase	AY575057	$(ATT)_{14}$	5′UTR	5′UTR–5′UTR	F: GCCCACCTCGTAACTTTGTACT<br>R: CTTCATGGGTGTCTTGTGTTGT	60	263	0, 237-293	-
RhDR01	dihydroflavonol 4-reductase	JF764809	$(TTGT)_3$	in2	in2–in2/ex3	F: TTTGTGTTTGAAGGAGCACATC<br>R: ATTGCTTACCCAACCAGTCATT	60	400	-	-
RhDR02	dihydroflavonol 4-reductase	JF764809	$(CTTCC)_2$	in1	ex1–in1/ex2	F: ATTCATCGGCTCTTGGTTTATG<br>R: CTTCACCTTCTTCTGATTCGCT	60	191	-	-
FaLR01	leucoanthocyanidin reductase	DQ834906	$(CAAAT)_2$	in2	ex2–in2	F: ATCACCCTAGTCGAAGCCATTA<br>R: GGTCATCAGTCCAAACACAGAA	60	138	0, 126-136	0, 134, 136
FaAS01	anthocyanidin synthase	AY695818	$(CTTGG)_2$	ex2	in–ex2	F: ATACGTTTTGCTAACTGGCGTC<br>R: CTGAGGGCATTTTGGGTAGTAG	60	211	207	-
RiAS01	anthocyanidin synthase	KX950789	$(ATCTC)_2$	ex2	ex2–ex2	F: TCAACAAGGAGAAGGTGAGGAT<br>R: CCGTTAGGAGAGATGAAAGCAG	60	334	261-333	261-357
FaAR01	anthocyanidin reductase	DQ664193	$(TGCTG)_2$<br>$(CATTT)_2$	ex4<br>in4	ex4–ex5	F: AATCTGCTTCTGGTCGGTACAT<br>R: AGAGAGTATGGTCTTCGCCTTG	60	244	227-242	250
RhAR01	anthocyanidin reductase	KR732618	$(GCTGC)_2$	ex5	ex5–ex5/in5	F: AATCTGCTTCTGGTCGGTACAT<br>R: GGTTAAAGGGAGGGCTTACTCA	60	122	-	-
FaFG01	flavonoid 3-O-glucosyltransferase	AY695816	$(TACG)_3$<br>$(CGC)_4$	5′UTR<br>ex1	5′UTR–ex1	F: CTGAAGAGTGGTTGTTGGATTG<br>R: TGCTTGGTGTTGAAGAAAGAGA	60	323	293-345	-

Locus	Gene	GenBank accession number	Motif and number of repeats	SSR location	Primer binding site location[1]	Forward (F) and reverse (R) primer nucleotide sequencies	T, Cº	Allele size, bp		
								expected	in *Fragaria*	in *Rubus*
RhUF01	UDP-glucose flavonoid 3-O-glycosyltransferase-like protein	JF764808	$(GAG)_7$ $(ACAAGC)_2$	ex2	ex2–ex2	F: AGGAGCTGAAGAAAAGACTCCA R: AAAGTCCTCTAGGTTTCCCCTG	60	275	-	267-279
FaMY01	transcription factor MYB10	EU155162	$(TTTAT)_2$ $(GT)_5$ $(TA)_6$ $(TA)_5$ $(AAATC)_2$	in2	in2–in2	F: CTACGGCTTATTCTTGCCTAGC R: TGTCAAACCTACACCGTTCAAG	60	383	0, 370-385	-
FaMY02	transcription factor MYB10	EU155162	$(AT)_{32}$-$(GT)_5$ $(TTTCT)_2$	in2	in2–ex3	F: TTGAACGGTGTAGGTTTGACAT R: TTCTTCACATCATTGGCAGTTC	60	310	0, 267-335	-
RiMY01	transcription factor MYB10	EU155165	$(TAATA)_2$ $(CT)_7$ $(AT)_{15}$	in1	ex1/in1–ex2	F: GTTCCTCTCCAAGCAGGTTATT R: TGCAAAGTCTCCTCTCTTGATG	59	330	206-239	0, 228-385
RiHL01	basic helix loop helix regulatory protein (bHLH)	EU862822	$(TACTA)_2$	in	ex1–-in	F: TTGCAGAGGAATGAGCAACTTA R: CAAACTATGGAACAAACGAGGA	60	336	291-299	286-338
RiTT01	transparent testa glabra 1 (TTG1) protein	HM579852	$(CAC)_5$	ex	ex–ex	F: ACTCCACACAAGAATCCCATCT R: CTGTTGTTCAAGACCGAAATTG	60	379	-	379, 382
FaCH01	class II chitinase	AF147091	$(CAAAA)_2$ $(CATCTT)_2$ $(TCTTCC)_2$	ex1	5′UTR–ex1	F: TGCTGATGCTAACTTCCTTTGA R: ATGCAGTGTCGTCCTTGTGTAG	60	324	310-329	0, 320-329
FaCH02	class II chitinase	AF147091	$(AATTAT)_2$ $(AGGCA)_2$ $(GAAGCC)_2$	ex2	in–ex2	F: TGAGAAATGGATGTTGTTCCAC R: AGCACATATCGACCTACCATGA	60	279	259-282	-
FaTG01	ToyoGluIII gene for β-1,3-glucanase	AB106362	$(TCAAGT)_2$	ex2	ex2–ex2	F: AGGATGAGGATTTATGAGCCAA R: AGGGTCCAAAAGGGTTGTATCT	60	327	309, 324	318-336
FaBG01	β-1,3-glucanase (BG2-2)	AY989818	$(GAGAG)_2$ $(GCGGGA)_2$ $(GA)_9$	5′UTR	5′UTR–5′UTR	F: TTACCTAGCTCAAGAGGGAGCA R: GTTTGGAAAGCCCACACTTTT	60	364	328-388	-

[1]based on the reference sequence; in – intron, ex - exon
